# Individual differences in chemotherapy-induced anticipatory nausea

**DOI:** 10.3389/fpsyg.2013.00502

**Published:** 2013-08-09

**Authors:** Marcial Rodríguez

**Affiliations:** Laboratory of Comparative Psychology, Department of Experimental Psychology, Faculty of Education and Humanities, University of GranadaCeuta, Spain

**Keywords:** chemotherapy, nausea, classical conditioning, differences, rat

## Abstract

Anticipatory Nausea (AN) is a severe side effect of chemotherapy that can lead cancer patients to discontinue their treatment. This kind of nausea is usually elicited by the re-exposure of the patients to the clinical context they need to attend to be treated. There has been considerable agreement that AN represents a paradigmatic example of Pavlovian conditioning, and within this framework, several behavioral interventions have been proposed in order to prevent this phenomenon. However, some studies have questioned the validity of the Pavlovian approach, suggesting that CS-US associations are neither necessary nor sufficient for AN to occur. The data and the alternative theories behind such criticisms are discussed. Additionally, it is suggested that animal models of AN could be enriched by taking into account rats' individual differences.

## Introduction

Chemotherapy treatment leads to a wide range of harmful collateral effects which include hair loss, diarrhoea, fatigue, loss of appetite, sexual dysfunction, and cognitive deficits (e.g., Kayl and Meyers, 2006). But in addition to these distressing side effects (perhaps to be expected given that, in essence, this treatment works through poisoning), the major unpleasant symptom that patients have to cope with while undergoing chemotherapy treatment is nausea (e.g., Haiderali et al., 2011). When severe, this consequence of chemotherapy dramatically reduces the patient's quality of life, and may even lead to discontinuation of the treatment (Roscoe et al., 2011). Adequate management of nausea for these patients has not been completely achieved through pharmacological interventions (Hsu, 2010), so that behavioral and cognitive therapies are being increasingly recommended (Schiff and Ben-Arye, 2011).

In an ordinary chemotherapy schedule (e.g., Jacobsen et al., 1993) high and low doses of cytotoxic drugs (such as cisplatin, carboplatin, cyclophosphamide) are administered in cycles spaced for a period of weeks. During each of those cycles, patients may need to attend the hospital for up to six consecutive weeks to receive an infusion on each visit. The visit to the hospital for the administration of the infusion can last for hours, and the first signs of intoxication (nausea, vomiting, sweating, changes in heart rate etc.) can be experienced when patients are still in the hospital room. Later, when they return home, sporadic nausea episodes can appear during the next 24 h, and also during a following period of ~5 days. These two phases are usually referred to as acute and delayed nausea, respectively (e.g., Haiderali et al., 2011). If patients undergoing chemotherapy repeatedly experience episodes of nausea, then this can lead to a further problem known as anticipatory nausea (AN).

## Pavlovian conditioning of nausea in cancer patients

In a given moment during the course of the treatment, cancer patients can experience nausea and/or vomiting before the start of a new infusion. Originally considered as a kind of neurosis, AN was finally identified in 1980 by Nesse et al. (1980) as a case of Pavlovian conditioning, an interpretation that prevails to the present day, albeit not exclusively. First, its etiology is taken to be psychological because this kind of nausea is not directly related to the infusion of the cytotoxic drugs; and secondly, it tends to occur when patients expect it on the basis of some specific environmental cues or thoughts. According to the classical conditioning model, the chemotherapy schedule can be conceptualized as a set of learning trials. Thus, in a particular context, the administration of the cytotoxic drugs would act as an unconditioned stimulus (US) with nauseating effects (the unconditioned response, UR). By virtue of association with the contextual stimuli present during the infusion sessions (conditioned stimulus, CS), these effects are subsequently elicited as a conditioned response (CR). The similarity between the UR and the CR; the fact that AN is more easy to observe as the chemotherapy treatment progresses (i.e., as the number of conditioning trials increases); that the stimuli acting as the CS are usually those related to the hospital setting (either directly perceived or imagined); and that AN persists during the follow-up visits to the hospital once the chemotherapy was completed, are four characteristics that clearly fit this interpretation (e.g., Tomoyasu et al., 1996).

The adequacy of a Pavlovian theoretical framework to account for AN has been widely accepted, and has had not only important implications for cancer patients but also for learning theorists. Patients have obtained two main benefits from the conditioning approach to AN. First, the simple fact of knowing the reasons why they react as they do has been supportive and a source of relief for them (Nesse et al., 1980). And second, two well-established learning phenomena that reduce the efficacy of CS-US pairings in producing an association—latent inhibition and overshadowing—have been offered (Stockhorst et al., 1998; Klosterhalfen et al., 2005) as possible behavioral interventions that could help to prevent AN^1^. Furthermore, this line of investigation has also been very fruitful for learning psychologists. Attempts to reproduce with laboratory animals the conditions under which AN develops in humans has helped to provide a useful paradigm for studying the laws of contextual aversion learning (see Symonds and Hall, 2012, for a review on this topic).

However, it is also necessary to recognize that there are two major problems that the Pavlovian framework needs to address. First, AN affects approximately only one in four patients (Roscoe et al., 2011), which means that factors other than CS-US contingencies may be affecting the development of AN, or in other words, that the predictive capacity of the Pavlovian model to identify those patients who are at risk of suffering from AN needs to be improved (Watson et al., 1998). Second, and more intriguingly, it has been asserted (Aapro et al., 2005) that nausea can be anticipated in patients without their having the previous experience that classical conditioning involves. In the following section we will first consider some data that do not fit with the conditioning model and the alternative theories that could account for them.

## Are alternative explanations for an necessary?

A good example of why some authors have questioned the validity of the associative theory as an account of AN was provided by Tyc et al. (1997). These authors observed that of the 45 children (59%) who developed AN in their sample, 11 (25%) had not previously suffered from post-treatment nausea (see also Matteson et al., 2002). This fact does not fit with learning rules in that the supposed CR could not be elicited if the subject has not previously experienced the UR. It might be possible to argue that experiencing the UR is not necessary for conditioning, i.e., that the simple fact of pairing the CS and the US could be enough for the formation of an association. However, such an argument could be considered as implausible and for the purposes of practical intervention in the clinic it seems reasonable to consider other possible explanations.

Tyc et al. (1997) proposed two possible accounts for their data. Firstly, nausea could have been directly elicited by an acute attack of anxiety, a phenomenon known as “psychogenic” or “nervous” nausea (Yugin, 1989). Secondly, it is known that a person can get sick through observational learning, i.e., by viewing other people vomiting. Given that subjects in the sample by Tyc et al., shared the chemotherapy room, this possibility seems more than plausible (see also Cohen et al., 1986). Finally, a third possibility, which has been increasingly analyzed during the last decade, is that the expectancies of the patients play an important role in the development of AN. Response expectancy theory supposes that patients might anticipate nauseating symptoms on the basis of their previous thoughts or beliefs and that this can be a direct cause of the occurrence of these symptoms (e.g., Montgomery et al., 1998; Sohl et al., 2009). In this case, AN would be governed by the same general mechanisms that operate in producing the placebo effect, and could be observed without the necessary mediation of a previous Pavlovian association (see Stewart-Williams and Podd, 2004, for a discussion about the relationship between the placebo effect and Pavlovian conditioning).

It is suggested then, that patients who expect to experience nausea, and even those who are uncertain about it, are more likely to develop AN than patients who clearly do not expect to get sick during the course of chemotherapy (Hickok et al., 2001). Given that the incidence of cancer among the population has increased over recent decades, patients may have acquired some knowledge from the media (through films, news, or documentaries), as well as from their friends and relatives, about the collateral side effects induced by the chemotherapy. The information provided by such unofficial sources, as well as that provided by medical staff, could influence the patient's expectations, thus producing a kind of “nocebo” effect (Colloca and Miller, 2011). Unfortunately, the few experimental attempts that have sought to confirm that the cancer patient's expectancies are a causal factor for nausea have reported inconsistent results. In one study, Shelke et al. (2008) showed that patients in an experimental group who trusted more than controls in the power of a new antiemetic medication, showed almost as much nauseating symptoms as the control. (Shelke et al., acknowledged, however, that their intervention may not have been enough to counteract the patient's previous expectancies, a possibility supported by the fact that the response expectancies assessed before the start of the experimental manipulation correlated with both the frequency and the severity of the post-treatment nausea). In contrast, Roscoe et al. (2010) did succeed in reducing the attacks of nausea by emphasizing the benefits of an acupressure technique in a previously identified “high-expectancy” group. But, unexpectedly, this manipulation also resulted in a significant augmentation of the occurrence of nausea in a group of patients who initially had low expectancies about it.

## Identifying an risk factors beyond CS-US contingencies

We should now turn to the apparent inability of the Pavlovian model to explain why some patients are at much higher risk of suffering AN than others. It has already been noted that, according to the contingency rules that govern the formation of associations, one might expect that all patients undergoing equivalent emetogenic chemotherapy schedules—i.e., a similar number of infusions with analogous cytotoxic drug doses—would suffer from similar AN symptoms. However, the validity of the contingency principle as the only factor that could account for AN is questioned by certain empirical findings. Firstly, it has been noted that the problem can occur under distinct chemotherapy regimes that, by employing the advised cytotoxic drugs, differ in their emetogenic capacity (van Komen and Redd, 1985; Andrykowski et al., 1988). In addition, in some cases no differences in the number of infusions, or in the severity of postchemotherapy nausea, have been found between those subjects who develop AN and those who do not (Fredrikson et al., 1993; Tyc et al., 1997). Finally, Andrykowski et al. (1988) noted that the consistency of AN was lower than would be expected on the basis of the Pavlovian model: in their study just 40% of patients who initially developed AN showed this response during the next 15 infusion sessions. Considering all of these facts, it seems necessary to accept that some variables other than those traditionally considered by learning theorists must be modulating the conditioning of nausea in cancer patients. Regression analyses have identified several factors, some of which can be classified as environmental or external, and others that refer to internal differences.

### External variables

It seems reasonable to assume that many environmental features might affect the capacity of the patients to cope with nausea. Certainly, the challenge that cancer patients must meet is severe and can push them almost to their limits. Under such extreme circumstances, it might be the case that some details that might otherwise be regarded as irrelevant could become much more significant in terms of managing the unwanted side effects. For example, several studies have pointed out that the family characteristics of the patients may help them to deal with the collateral emetic effects of chemotherapy. Patients who have, for instance, non-conflicting and balanced families that allow them to speak openly about their suffering are less likely to experience AN (see, e.g., Youngmee and Morrow, 2007). In addition, Cohen et al. (1986) found that the characteristics of the treatment center strongly predicted the presence of AN, and suggested that some fine points pertaining to the chemotherapy room, such as being within sight of basins or the absence of entertainment or comfortable chairs, could facilitate the manifestation of emetic anticipatory symptoms. Further support for this suggestion comes from recent studies (e.g., McCarthy et al., 2012) claiming that cozy waiting and treatment rooms are necessary to reduce both anticipatory anxiety and pain in cancer patients.

Another hospital-related difference affecting patients is the antiemetic protocol dispensed by nurses and doctors. Clearly this practice is likely to be important as it constitutes the first pharmacological line of defense against the emetic syndrome induced by the chemotherapy. It is, however, far from uniform and the unification of intervention protocols still remains an unreached goal (e.g., Schwartzberg, 2011). Furthermore, it has been asserted that antiemetic treatments are often incorrectly employed by doctors and nurses (e.g., Burmeister et al., 2012; Fernández-Ortega et al., 2012), perhaps because medical staff fail to appreciate fully the severity of the symptoms (Foubert and Vaessen, 2005; Majem et al., 2011). Given the close relationship between post-treatment nausea and AN, and that reports on this topic often recruit their sample from different hospitals, the adequacy of the antiemetic intervention can be an important factor in generating differences in AN. In this regard it should be noted that such variations could be argued by associative theorists to explain why similar chemotherapy regimes do not always produce equivalent anticipatory symptoms. An adequate use of this prophylactic medication could avoid, at least in some degree, the emetic capacity of chemotherapy and hence would reduce the possibilities of nausea conditioning. Thus, in a study including patients from different hospitals, a similar number of infusions of equivalent cytotoxic drugs should only be taken as a comparable contingency program if there are evidences that the antiemetic protocol is also equivalent.

### Internal variables

Personality variables are known to affect conditioning in humans but, in spite of this, they are not usually theoretically integrated into traditional associative learning models. It seems reasonable, particularly from a practical point of view, to know if cancer patients who develop AN do share some characteristics. Thus, in addition to hospital and family differences, regression models have isolated some other variables that provide a profile of those patients who are at greatest risk of suffering from AN. These studies indicate that variables such as being less than 50 years old, having susceptibility to motion sickness or nausea during pregnancy, and being under a state of anxiety, hostility or depression, can account for part of the variability of the occurrence of AN (e.g., Roscoe et al., 2011). This risk profile can be extended by taking into account some personality traits associated with AN. For instance, Challis and Stam (1992) found that AN correlated with higher scores in scales measuring suggestibility, and van Komen and Redd (1985) have reported similar correlations with traits such as future despair, social alienation, inhibited personality style, and anxiety. Additionally, Hursti et al. (1992) asked relapse-free cancer patients to complete several personality scales and to report how they experienced nausea when attending chemotherapy. Their results showed that neuroticism and inhibiting style were two dimensions that correlated with AN. Interestingly, this same sample of subjects was further explored by Fredrikson et al. (1993) in an attempt to determine if AN patients were more susceptible to conditioning. Subjects in this sample were classified as AN or Non-AN, and treated as independent groups. Their capacity to associate visual figures with a mild electric shock was assessed using a heart-rate measure. They found that patients who developed AN were more easily conditioned than those in the Non-AN group. Taken together, these latter two studies suggest a relationship between personality traits (a high score in neuroticism or introversion) and the aversive conditioning of both nausea and fear. This conclusion, however, needs to be treated with caution. First, the groups were compared as if their distribution was randomized when it was not, and secondly, the possibility exists that in a retrospective study of this sort, the effects were generated by the chemotherapy treatment itself.

## The use of individual differences in animal models of AN

The difficulties of carrying out an experimental assessment of the role of personality traits in the development of AN might be resolved by using an animal model. Individual differences are not only to be found in our species—several studies have demonstrated consistent individual differences in rats and, moreover, that some of these can be used to predict some Pavlovian related phenomena (e.g., Robinson and Flagel, 2009). Of course there are features of human personality that cannot be modeled in animals as they are exclusively revealed as verbal thoughts. However, other animal behaviors parallel reasonably well some individual characteristics that, as it has just been mentioned, are present in those cancer patients that develop AN.

On the other hand, contextual aversions modeling AN can easily be reproduced in the rat by simply pairing a novel environment with the effects of an emetic drug. Rodríguez et al. (2000) showed that exposures to a novel place following an injection of lithium chloride (a fast-acting emetic drug) produce a learned aversion that can be assessed by simply measuring the ingestion of a novel flavor offered in that place—after a few of those trials a novel palatable solution is consumed unwillingly. The aversive properties acquired by the context after such training have been evaluated through other more accurate aversive measures such as the taste reactivity test (e.g., Limebeer et al., 2006), supporting the validity of the model. In the next sections the possibilities of using some animal differences to improve the modeling of AN will be discussed.

### Animal differences in anxiety

Traits labeled anxious-neurotic in humans can be assessed in rats by using tests such as the elevated maze or defensive burying behavior. In particular, a reluctance to enter open arms, or a failure to cover dangerous or disgusting objects, can be considered as a sign that a rat is anxious (Ho et al., 2002). A possible way of testing the influence of personality traits on Pavlovian conditioning of nausea, therefore, could be to assess if anxious rats show better acquisition of a context-illness association.

To our knowledge this specific investigation has not yet been carried out, but there are some studies that support its viability. Borta et al. (2006), for example, observed that rats with a low tendency to enter open arms, i.e., those supposed to be more anxious, seemed to learn more readily an association between a tone and a shock. In another study, Walker et al. (2008) found that results obtained in the test of defensive burying behavior predicted stronger aversive learning in which a new cage (a contextual CS) was paired with attacks by a male (bites as the US) living in that context:44% of the variability in locomotor activity in that cage during the test (carried out when the resident male was not present) was explained by the previous defensive burying related behaviors that the rats showed in response to prods that had been placed in their home cages.

The role of anxiety differences in the success of associative interventions intended to prevent AN could also be analyzed using animal models. Latent inhibition and overshadowing (see Note 1), which have already been shown to reduce AN in rats (see Symonds and Hall, 2012), demand attentional processes (e.g., Granger et al., 2012) that could be affected by a state of acute anxiety (Braunstein-Bercovitz et al., 2002). If anxiety disrupts the capacity to select those more reliable environmental stimuli, the retardation in aversive context conditioning derived from these interventions could be in question for these more anxious subjects. This can be analyzed by testing if, in a preexposed or overshadowed context, anxious rats consume less of a novel taste than normal rats.

### Other animal differences

Regressive methodology can also readily be used to assess, in a highly controlled way, the relationship between experimentally induced AN and other individual characteristics, even though, unlike anxiety, these are not facilitators of aversive conditioning in non-human animals. For example, the application of unpredictable chronic mild stress (UCMS) in rats to model human depression has no effect on contextual aversion but, interestingly, it does impair place preference. In particular, UCMS appears to produce anhedonia, i.e., a reduction in the capacity of the subjects to appreciate the appetitive effects of the rewarding drugs. (see Willner and Mitchell, 2002 for a review of these models.)

Anhedonia is a core symptom of human depression that can be identified in rats by simply registering its ingestion of sucrose (e.g., Strekalova and Steinbusch, 2010; but see Matthews et al., 1995). Thus, a possible investigation to test if depression is related to AN could be to analyze if subjects displaying a lower preference for sucrose are later more likely to develop AN. Similarly, the connection between hostility and AN could be studied in animals by using some rat strains that differ in their latency to attack an opponent male—the shorter the latency of attack the higher the supposed level of aggression (e.g., de Boer et al., 2003). Considering these examples, it seems promising to use this methodological strategy to complete a better model of AN, by seeing whether any other differential characteristic present in the AN population can also be validated in rodents.

## Conclusion

It is widely acknowledged that animal models can be a useful first step in testing the validity of any novel antiemetic intervention. And it is now clear that developing animal models of AN that take into account individual differences will bestow certain advantages. First, it will clearly be more efficient to test the reliability of any given prophylactic intervention in animals known to have a particular sensitivity to the conditioning of nausea. And success in identifying the personality variables that make some people particularly vulnerable to AN would then allow us to focus our antiemetic efforts on such people, providing them with more accurate treatment that is appropriate for their specific profile. For example, relaxation techniques may be necessary in those patients with high scores in anxiety before the application of associative interventions in order to guarantee its maximum efficiency. The costs involved in pharmaceutical and psychosocial interventions are substantial, providing a further reason for concentrating our efforts on these more vulnerable patients and giving them more adequate therapy. Finally, such studies could pay theoretical dividends by confirming the relevance of certain traits or response tendencies as predictors of the development of AN.

### Conflict of interest statement

The author declares that the research was conducted in the absence of any commercial or financial relationships that could be construed as a potential conflict of interest.

## Footnotes

^1^Latent inhibition is a very robust effect that has been observed under a great variety of preparations—presentations of a stimulus followed by no consequences will subsequently retard the acquisition of its association with any given US. Klosterhalfen et al. (2005) showed for example that AN induced by a rotation chair was lower in subjects preexposed to that apparatus. Thus, within the context of chemotherapy treatment, these authors suggested that preexposures to the clinical cues could be useful to prevent chemotherapy-induced AN. Overshadowing is also a well-established phenomenon in associative learning—a salient cue is presented in combination with a target CS, with the result that the capacity of that CS to predict the US is reduced. Stockhorst et al. (1998) gave a group of cancer patients different novel combinations of flavors during their first two infusions. At their third clinical visit none of the 8 experimental subjects showed AN, but it was found in two patients of the control group that had drunk water instead of the novel tastes. It is supposed that the readiness of the flavors to become associated with the gastric discomfort overshadowed the conditioning of the clinical cues.
